# The impact of muscular atrophy on functional outcomes in pediatric critical care

**DOI:** 10.62675/2965-2774.20260250

**Published:** 2026-01-14

**Authors:** Esteffany Carvalho de Fraga, Jéssica Knisspell de Oliveira, Taila Cristina Piva, Renata Salatti Ferrari, Ian Teixeira e Sousa, Francisco Bruno, Camila Wohlgemuth Schaan, Janice Luisa Lukrafka

**Affiliations:** 1 Universidade Federal de Ciências da Saúde de Porto Alegre Department of Physiotherapy Porto Alegre RS Brazil Department of Physiotherapy, Universidade Federal de Ciências da Saúde de Porto Alegre - Porto Alegre (RS), Brazil.; 2 Universidade Federal de Ciências da Saúde de Porto Alegre Porto Alegre RS Brazil Postgraduate Program in Pediatrics in Child and Adolescent Care, Universidade Federal de Ciências da Saúde de Porto Alegre - Porto Alegre (RS), Brazil.; 3 Universidade Federal do Rio Grande do Sul Hospital de Clínicas de Porto Alegre Department of Physiotherapy Porto Alegre RS Brazil Department of Physiotherapy, Hospital de Clínicas de Porto Alegre, Universidade Federal do Rio Grande do Sul - Porto Alegre (RS), Brazil.; 4 Hospital da Criança Conceição Grupo Hospitalar Conceição Porto Alegre RS Brazil Hospital da Criança Conceição, Grupo Hospitalar Conceição - Porto Alegre (RS), Brazil.; 5 Universidade Federal do Rio Grande do Sul Hospital de Clínicas de Porto Alegre Department of Pediatric Intensive Care Porto Alegre RS Brazil Department of Pediatric Intensive Care, Hospital de Clínicas de Porto Alegre, Universidade Federal do Rio Grande do Sul - Porto Alegre (RS), Brazil.

Acquired muscular weakness in pediatric critical patients may affect functional recovery.^(1)^ Patients with poorer functional outcomes showed a higher readmission rate to the pediatric intensive care unit (ICU),^(2)^ and children with poorer long-term functional outcomes are more likely to require mechanical ventilation, use vasoactive drugs, and have prolonged pediatric ICU stay.^(3)^ However, the factors contributing to a decline in functional status remain poorly understood. This study aimed to assess the association between the occurrence of muscular atrophy and the development of new morbidity at pediatric ICU and hospital discharge.

This prospective study was conducted at a public hospital in Southern Brazil and was approved in accordance with national guidelines (CAAE: 46901521.7.0000.5327).

It included children aged 1 month to 12 years, who had undergone mechanical ventilation for at least 24 hours. We excluded those who were dependent on ventilatory technology before admission to the pediatric ICU, readmitted to the pediatric ICU < 24 hours after discharge, or had a neurological/genetic diagnosis associated with muscle weakness/muscle tone. Clinical and demographic information was collected from electronic medical records. The patients were categorized into two groups: "respiratory diagnosis" category and "other diagnoses".

The muscle thickness was measured using a portable US imaging device, Sonosite M-turbo (FUJIFILM Sonosite, WA, USA), with a 6-13MHz linear probe, and the measurements were conducted following previously published protocols from our group.^(4)^ The brachial biceps/brachialis and rectus femoris/vastus medialis were examined at the following: up to 24 hours on the first day of admission, after 72 hours, and weekly until discharge from the pediatric ICU, as long as it did not exceed a period longer than 28 days in the unit. Muscle atrophy was defined as a reduction of 10% or more in muscle thickness (last-first assessment).^(5)^

The functional assessment was performed using the validated FSS-Brazil.^(6,7)^ Patients were assessed at the pediatric ICU and hospital discharge, and pre-hospitalization scores were retrospectively obtained by parent recall.^(3)^ The emergence of new morbidity was defined as a two-point or more increase in a single domain compared to the pre-hospitalization score.^(8)^

The association between new morbidity and muscular atrophy was verified using Fisher's Exact Test. A significance level of 5% (p < 0.05) and Statistical Package for Social Science (SPSS) version 26.0 for Windows, for all analyses.

A total of 101 patients were included, the majority male, with a mean age of 6 months. The prevalence of muscle atrophy was 39.6% and at the time of pediatric ICU discharge, the prevalence of new morbidity was 77.2% (Table 1). The prevalence of new morbidity was significantly higher in patients with atrophy compared to those without atrophy among the group with other diagnoses at the time of pediatric ICU discharge (Figure 1).

**Table 1:** Sample demographic and clinical characteristics

Variable			
Age (months)			6 (2 - 24)
Sex (male)			64 (63.4)
Time on mechanical ventilation (days)			4 (3 - 7)
Length of stay on pediatric ICU (days)			8 (5 - 12)
Total length of stay (days)			35.99 (6 - 222)
Muscular atrophy			40 (39.6)
New morbidity at pediatric ICU discharge			78 (77.2)
New morbidity at hospital discharge			23 (22.8)
	Diagnosis		
		Respiratory	71 (70.3)
		Others*	30 (29.7)
Death			10 (9.9)
Extubation failure, yes			10 (9.9)
Pediatric ICU-free days			7 (0 - 15.5)
Ventilator-free days			20 (16 - 23)

ICU - intensive care unit.

* Gastrointestinal, hepatic, oncological, metabolic, genetic, cardiovascular, renal, and others. Results are expressed as median (interquartile range) or n (%).

**Figure 1:**
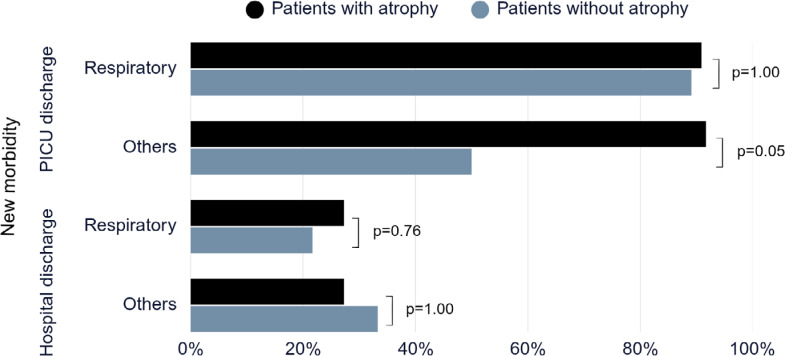
Association between atrophy (yes or no) and who developed new morbidity at the pediatric intensive care unit and hospital discharge.

ICU - intensive care unit.

In children with respiratory diagnoses, muscle mass loss was not associated with worse functional outcomes. However, in patients with other diagnoses, the onset of new morbidity at pediatric ICU discharge was related to the occurrence of muscle atrophy. Probably, patients with respiratory diseases are previously healthy, manifest an acute critical condition, but experience rapid recovery. On the other hand, patients in the other group represent a profile of more chronic diseases and prolonged treatments. Moreover, the prevalence of new morbidity decreased to less than half at the time of hospital discharge, indicating the recovery of children after the ICU stay. Differences in age and motor development (walking or non-walking) could impact the relationship between muscle mass loss and functional status and need further investigation, such as a rehabilitation program implemented during hospitalization. Additionally, it is a single-center study, reducing the generalizability to other populations.

We showed an association between muscle atrophy and the development of new morbidity at pediatric ICU discharge only in children without respiratory diagnoses.

## Data Availability

Data is available on demand from referees.

## References

[1] Siu K, Al-Harbi S, Clark H, Thabane L, Cheng J, Tarnopolsky M (2015). Feasibility and reliability of muscle strength testing in critically ill children. J Pediatr Intensive Care.

[2] Pereira GA, Schaan CW, Ferrari RS (2017). Functional evaluation of pediatric patients after discharge from the intensive care unit using the Functional Status Scale. Rev Bras Ter Intensiva.

[3] Pinto NP, Rhinesmith EW, Kim TY, Ladner PH, Pollack MM (2017). Long-term function after pediatric critical illness: results from the survivor outcomes study. Pediatr Crit Care Med.

[4] de Oliveira JK, Schaan CW, Silva CK, Piva TC, Sousa IT, Bruno F (2023). Reliability of ultrasound in the assessment of muscle thickness in critically ill children. An Pediatr (Engl Ed).

[5] Johnson RW, Ng KWP, Dietz AR, Hartman ME, Baty JD, Hasan N, Zaidman CM, Shoykhet M (2018). Muscle atrophy in mechanically ventilated critically ill children. PLoS ONE.

[6] Pereira GA, Schaan CW, Ferrari RS, Normann TC, Rosa NV, Ricachinevsky CP (2019). Functional Status Scale: cross-cultural adaptation and validation in Brazil. Pediatr Crit Care Med.

[7] Pollack MM, Holubkov R, Glass P, Dean JM, Meert KL, Zimmerman J (2009). Eunice Kennedy Shriver National Institute of Child Health and Human Development Collaborative Pediatric Critical Care Research Network. Functional Status Scale: new pediatric outcome measure. Pediatrics.

[8] Pollack MM, Banks R, Holubkov R, Meert KL, the Eunice Kennedy Shriver National Institute of Child Health and Human Development Collaborative Pediatric Critical Care Research Network (2021). Long-term outcome of PICU patients discharged with new, functional status morbidity. Pediatr Crit Care Med.

